# Impacts of recent cultivation on genetic diversity pattern of a medicinal plant, *Scutellaria baicalensis *(Lamiaceae)

**DOI:** 10.1186/1471-2156-11-29

**Published:** 2010-04-29

**Authors:** Qing-Jun Yuan, Zhi-Yong Zhang, Juan Hu, Lan-Ping Guo, Ai-Juan Shao, Lu-Qi Huang

**Affiliations:** 1 Laboratory for Molecular Pharmaceutics, Institute of Chinese Materia Medica, China Academy of Chinese Medical Sciences, Beijing 100700, China; 2 Laboratory of Subtropical Biodiversity, Jiangxi Agricultural University, Nanchang, Jiangxi 330045, China

## Abstract

**Background:**

Cultivation of medicinal plants is not only a means for meeting current and future demands for large volume production of plant-based drug and herbal remedies, but also a means of relieving harvest pressure on wild populations. *Scutellaria baicalensis *Georgi (Huang-qin or Chinese skullcap) is a very important medicinal plant in China. Over the past several decades, wild resource of this species has suffered rapid declines and large-scale cultivation was initiated to meet the increasing demand for its root. However, the genetic impacts of recent cultivation on *S. baicalensis *have never been evaluated. In this study, the genetic diversity and genetic structure of 28 wild and 22 cultivated populations were estimated using three polymorphic chloroplast fragments. The objectives of this study are to provide baseline data for preserving genetic resource of *S. baicalensis *and to evaluate the genetic impacts of recent cultivation on medicinal plants, which may be instructive to future cultivation projects of traditional Chinese medicinal plants.

**Results:**

Thirty-two haplotypes of *S. baicalensis *(HapA-Y and Hap1-7) were identified when three chloroplast spacers were combined. These haplotypes constituted a shallow gene tree without obvious clusters for cultivated populations, suggesting multiple origins of cultivated *S. baicalensis*. Cultivated populations (*h*_T _= 0.832) maintained comparable genetic variation with wild populations (*h*_T _= 0.888), indicating a slight genetic bottleneck due to multiple origins of cultivation. However, a substantial amount of rare alleles (10 out of 25 haplotypes within wild populations) lost during the course of *S. baicalensis *cultivation. The genetic differentiation for cultivated group (*G*_ST _= 0.220) was significantly lower than that of wild group (*G*_ST _= 0.701). Isolation by distance analysis showed that the effect of geographical isolation on genetic structure was significant in wild populations (*r *= 0.4346, *P *< 0.0010), but not in cultivated populations (*r = *0.0599, *P *= 0.2710). These genetic distribution patterns suggest that a transient cultivation history and the extensive seed change among different geographical areas during the course of *S. baicalensis *cultivation.

**Conclusions:**

Although cultivated *S. baicalensis *maintains comparable genetic diversity relative to wild populations, recent cultivation has still imposed profound impacts on genetic diversity patterns of the cultivated *S. baicalensis *populations, i.e., the loss of rare alleles and homogenization of cultivated populations. This study suggests that conservation-by-cultivation is an effective means for protecting genetic resources of *S. baicalensis*, however, the wild resources still need to be protected *in situ *and the evolutionary consequences of extensive seed exchange mediated by human being should be monitored carefully.

## Background

The World Health Organization has estimated that more than 80% of the world's population depends on herbal medicine for primary healthcare needs [1]. Most materials used in herbal medicine and vitamin supplements are taken from wild plants and the rapid growing demands for medicinal plants, compounded by habitat loss, is exerting pressure on many species. With the increased realization that some wild species are being over-exploited, a number of governments and agencies are recommending that wild medicinal species should be brought into cultivation systems [2]. Cultivation of medicinal plants is not only a means for meeting current and future demands for large volume production of plant-based drug and herbal remedies, but also a means of relieving harvest pressure on wild populations [3,4]. In certain circumstances such as traditional agriculture, cultivation can serve as an important reservoir of genetic variability [5,6].

However, cultivation can also have impacts on conservation, which need to be better understood [4]. Medicinal plant production through cultivation, for example, can reduce the incentives to conserve wild populations [7]. More importantly, founder effects and artificial selections for high yielding or pharmacologically standard individuals would probably result in a narrow genetic range of material in cultivation, resembling the genetic bottleneck during the domestication of cereal species [8]. Furthermore, seed exchange between farmers is much easier due to the highly developed transportation and commercial markets of modern times [9], but migration of seeds from their collection sites to other environments within a species range for cultivation may increase the risk of maladaptation [10,11]. Gene flow from maladapted cultivated individuals into adjacent native populations could also negatively affect adaptation to local environments [12].

Medicinal plants play inherent and prominent roles in the general health service in China. Due to long-term exploitation of wild medicinal herbs, many important Chinese traditional medicinal plants are becoming rare and endangered [13]. In order to protect the medicinal plant resources and meet the increasing demand for plant-based drug and herbal remedies, the most popular medicinal plants have been cultivated under the supervision of Chinese government or grown spontaneously by farmers [13]. Today, the cultivation areas of medicinal plants reach 5 million mus (1 mu = 0.165 acre) [14]. The sharp increase in medicinal plant cultivation may greatly mitigate the pressure on the wild medicinal resources, but also impose profound impacts on the genetic diversity patterns of these medicinal plants. However, studies about genetic impacts of cultivation are mostly concentrated on economically valuable crops with long domestication history (see review in [8]), little is known regarding the genetic impacts of recent cultivation on medicinal plants (but see [15-18]). This issue becomes more important as wild gene pools of many medicinal species have decreased rapidly or become extinct. Domestic cultivation can not only meet the increasing demands of human consumption [15], but also represent a conservation-through-use approach for protecting genetic resources of medicinal plants [16].

*Scutellaria baicalensis *Georgi (Huang-qin or Chinese skullcap) is a perennial herb of the family Lamiaceae with fleshy root, branched stems, papery leaves, purple-red to blue flowers, and black-brown ovoid nutlets [19]. Root of this herb (Radix Scutellariae) is a very important Chinese Materia Medica that was first recorded in *Shen Nong Ben Cao Jing *in *ca*. 100 BC [20] and officially listed in the Chinese Pharmacopoeia [21]. As one of the most important heat-clearing and dehumidifying drugs in traditional Chinese medicine, Radix Scutellariae has been widely used in the treatment of hepatitis, jaundice, tumor, leukemia, hyperlipaemia, arteriosclerosis, diarrhea, and inflammatory diseases [22-24]. Huang-qin is pollinated by bees and flies, and propagated by seeds [25]. The wild populations of *S. baicalensis *is widely distributed in Nei mongol, Heilongjiang, Jilin, Liaoning, Hebei, Shandong, Jiangsu, Henan, Hubei, Shanxi, Shaanxi, and Gansu provinces from Northeast to Northwest China and adjacent areas including Mongolia, Russia, Korea and Japan [19]. This species often appears in grassland, sunny grassy slopes and shrubbery or forest habitats (from 12 to 2000 m) with a cold-dry climate [19]. Over the past several decades, the wild resource of *S. baicalensis *has suffered rapid declines, and thus was listed as a class III conserved medicinal plant in China [26]. To meet the increasing demand for its root, large-scale cultivation programs have been initiated in China since 1958 [27]. Because *S. baicalensis *is a newly-cultivated species and there are still plenty of wild populations, this medicinal species may represent an ideal model to evaluate the impacts of recent cultivation on the genetic diversity pattern of medicinal plants. Three polymorphic chloroplast fragments were chosen for population genetic analyses in this study because chloroplast markers are more sensitive than nuclear markers to demographic fluctuations such as cultivation bottlenecks resulting from smaller effective population sizes (*N*_e_) [28]. In addition, chloroplast markers are powerful tools for tracing the maternal origins of cultivated populations and seed exchange among populations, which are of important relevance to genetic resource conservation [6,29]. The objectives of this study were to: 1) quantify the amount of genetic variation in wild and cultivated populations of *S. baicalensis*; 2) compare the genetic structure in wild and cultivated populations of *S. baicalensis*; 3) evaluate the impacts of recent cultivation on the genetic diversity pattern of *S. baicalensis*; and 4) unravel the meaning of recent cultivation on medicinal plant germplasm conservation.

## Methods

### Sampling

**Figure 1:**
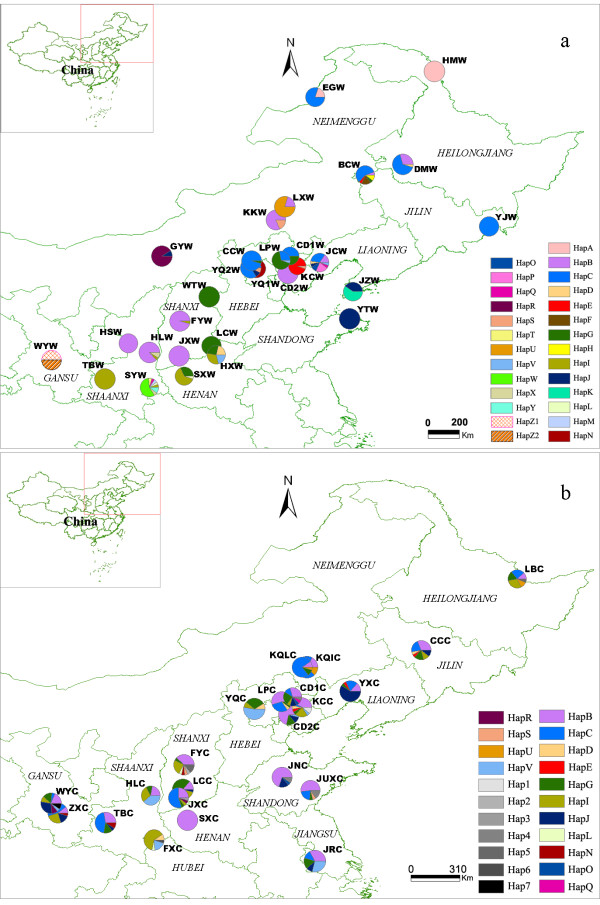
**Geographic distribution and frequencies of chloroplast haplotypes in wild *Scutellaria baicalensis *and *Scutellaria rehderiana *(WYW) (a) and cultivated *Scutellaria baicalensis *(b) populations**. Population abbreviations are the same as Table 1. The pie sizes of populations are proportional to their sample sizes.

A total of 602 and 451 individuals of *S. baicalensis *representing 28 wild and 22 cultivated populations were collected from Northeast to Northwest China, respectively (Table 1, Fig. 1). Seventeen to twenty-four individuals were sampled for each population. Twenty-two *Scutellaria rehderiana *Diels individuals were sampled from a wild population of Weiyuan county in Gansu province (WYW) as an outgroup (Table 1, Fig. 1a). Leaves for DNA extraction were dried with silica gel. Voucher specimens were deposited in herbaria of Institute of Chinese Materia Medica (CMMI), China Academy of Chinese Medical Sciences. The precise geographic location of each sampled population was determined using a Garmin GPS unit (Table 1).

**Table 1:** Details of sample locations, sample sizes in 28 wild and 22 cultivated populations of *Scutellaria baicalensis *and 1 wild population of *Scutellaria rehderiana*.

Province	County	P	**Lat**.	**Long**.	Alt.(m)	N
Neimenggu	Eerguna	EGW	50.42°	119.51°	564.7	20
Neimenggu	Linxi	LXW	44.05°	117.76°	1212	24
Neimenggu	Keshiketeng	KKW	43.28°	117.23°	1399	22
Neimenggu	Guyang	GYW	41.20°	110.60°	1854	24
Neimenggu	Kalaqin	KQLC	42.90°	118.50°	691.2	20
Neimenggu	Kalaqin	KQIC	42.90°	118.76°	691.2	24
Heilongjiang	Huma	HMW	51.93°	126.43°	288.3	24
Heilongjiang	Duerbote	DMW	46.51°	124.59°	146.2	24
Heilongjiang	Luobei	LBC	47.91°	130.72°	184.2	17
Jilin	Baicheng	BCW	45.90°	122.42°	240.8	19
Jilin	Yanji	YJW	42.92°	129.60°	302.1	21
Jilin	Changchun	CCC	43.87°	125.27°	241	19
Liaoning	Jianchang	JCW	40.82°	119.78°	362	22
Liaoning	Jinzhou	JZW	39.13°	121.70°	12	20
Liaoning	Yixian	YXC	41.53°	121.23°	61	22
Hebei	Chengde	CD1W	41.20°	117.94°	58	18
Hebei	Chengde	CD1C	41.20°	117.94°	58	17
Hebei	Chengde	CD2W	40.20°	117.94°	60	23
Hebei	Chengde	CD2C	40.20°	117.73°	60	21
Hebei	Kuancheng	KCW	40.62°	118.47°	304	17
Hebei	Kuancheng	KCC	40.61°	118.49°	304	19
Hebei	Luanping	LPW	40.95°	117.53°	523	18
Hebei	Luanping	LPC	40.93°	117.33°	523	20
Hebei	Chicheng	CCW	40.92°	115.82°	1100	22
Beijing	Yanqing	YQ1W	40.47°	115.97°	900	19
Beijing	Yanqing	YQ2W	40.52°	115.78°	1300.6	22
Beijing	Yanqing	YQC	40.52°	115.78°	600	23
Shandong	Yantai	YTW	37.55°	121.52°	25	24
Shandong	Jinan	JNC	36.64°	117.36°	200	20
Shandong	Juxian	JUXC	35.90°	118.97°	179	19
Jiangsu	Jurong	JRC	31.87°	119.22°	22	24
Henan	Huixian	HXW	35.46°	113.77°	863	22
Henan	Songxian	SXW	34.22°	111.91°	864.5	19
Henan	Songxian	SXC	34.18°	111.97°	539.3	21
Hubei	Fangxian	FXC	33.06°	110.07°	918	21
Shanxi	Wutai	WTW	38.83°	113.36°	1148	24
Shanxi	Fenyang	FYW	37.42°	111.65°	1588	23
Shanxi	Fenyang	FYC	37.35°	111.77°	947	21
Shanxi	Lingchuan	LCW	35.98°	113.49°	1406	21
Shanxi	Lingchuan	LCC	35.95°	111.72°	1363	22
Shanxi	Jiangxian	JXW	35.39°	111.61°	854.5	23
Shanxi	Jiangxian	JXC	35.47°	111.46°	545	20
Shaanxi	Huanglong	HLW	35.59°	109.88°	1218	24
Shaanxi	Huanglong	HLC	35.59°	109.88°	1160	18
Shaanxi	Shanyang	SYW	33.56°	109.89°	949.0	19
Shaanxi	Taibai	TBW	34.06°	107.30°	1682	24
Shaanxi	Taibai	TBC	34.04°	107.30°	1565	22
Gansu	Heshui	HSW	36.12°	108.67°	1121	20
Gansu	Weiyuan	WYW*	35.15°	104.21°	2115	22
Gansu	Weiyuan	WYC	35.15°	104.21°	2090	21
Gansu	Zhangxian	ZXC	34.60°	104.60°	2065	20

Abbreviations: P, population code; Lat., latitude; Long., longitude; Alt., altitude; N, number of sampled individuals; the last letter of population code: W, wild; C, cultivated; *: *Scutellaria rehderiana*.

### DNA extraction, amplification and sequencing

Dried leaves were milled by using RETSCH Mixer Mill (MM301). Genomic DNA was extracted using a modified cetyltrimethyl ammonium bromide (CTAB) protocol [30]. The universal primers described in Hamilton [31] and Sang *et al*. [32] were used for screening polymorphic cpDNA fragments. After preliminary screening of eight chloroplast fragments, we chose *atp*B-*rbc*L, *trn*L-*trn*F and *psb*A-*trn*H intergenic spacers for the full survey because they contained the most polymorphic sites. PCR amplification was performed in a TC-512 thermocycler (Techne, England) programmed for an initial 240 s at 94°C, followed by 30 cycles of 45 s at 94°C, 30 s at 55°C (*atp*B-*rbc*L), 54°C (*trn*L-*trn*F) or 58°C (*psb*A-*trn*H), 90 s at 72°C, and a final 240 s at 72°C. Reactions were carried out in a volume of 20 μL containing 2.0 mm/L MgCl_2_, 0.5 μm/L dNTP, 10 × buffer, 2.5 μm/L primer, 1 U *Taq *DNA and 20 ng DNA template. Sequencing reactions were conducted with the forward or reverse primers of the PCR using the DYEnamic ET Terminator Kit (Amersham Pharmacia Biotech), following the manufacturer's protocol. Sequencing was performed on a HITACHI 3130 Genetic Analyzer (Hitachi High-Technologies Corporation, Tokyo Japan) after the reaction product was purified through precipitation with 95% ethanol and 3 M sodium acetate (pH 5.2).

### Data analysis

Sequences were aligned using Clustal_X version 1.81 [33], and all indels were coded as substitutions following Caicedo and Schaal [34]. All individuals were characterized for cpDNA haplotype. Sequence variation was tested for deviations from neutrality by Tajima's *D *statistic [35], and by Fu and Li's D* and F* statistics [36] using DNASP 4.00 [37]. A haplotype network depicting the mutational relationships among distinct haplotypes was drawn following the principle of parsimony by TCS version 1.13 [38,39], positing *S. rehderiana *as outgroup. The geographical distribution of haplotypes was plotted on a map of China using Arcmap 8.3 (ESRI, Inc.). Significant difference of haplotype frequencies between wild and cultivated populations was quantified using *x*^2 ^test. Total diversity (*h*_T_), within-population diversity (*h*_S_) and level of population differentiation (*G*_ST_) were calculated using the program HAPLONST, treating wild and cultivated populations as separate groups. The significance of the parameter comparisons (Number of haplotype; Total diversity, *h*_T_; Within-population diversity, *h*_S_; Population differentiation, *G*_ST_) between wild and cultivated groups were estimated by *x*^2 ^test and Wilcoxon two-group tests, being implemented in the statistical package SPSS17.0 (SPSS Inc.).

Hierarchical structure of genetic variation in *S. baicalensis *was determined by an analysis of molecular variance (AMOVA) with ARLEQUIN version 2.000, partitioning genetic variance into three levels: among groups (cultivated and wild groups), among-population within groups, and within-population. Genetic differentiation between cultivated and wild groups was also evaluated using DNASP 4.00 [37]. To examine the effect of geographical distance on genetic structure, correlations between pairwise genetic distances (Kimura 2-parameter distance generated with Kimura's two parameter model in MEGA 3) [40-42] and pairwise geographic distances were tested using a Mantel test implemented by Isolation By Distance Web Service [43].

## Results

### Sequence characteristics of three chloroplast intergenic spacers

The aligned sequences of *atp*B-*rbc*L, *trn*L-*trn*F, and *psb*A-*trn*H spacers in *S. baicalensis *and *S. rehderiana *were 768, 799 and 502 base pairs in length, respectively. There were 13, 7, and 14 polymorphic sites (including substitutions and indels) for the three spacers, respectively (Table 2). Because sequencing poly-N regions could easily lead to homoplasies due to polymerase error [44], the short sequence repeats (poly-C) between 502-508 bp of *trn*L-*trn*F were not treated as polymorphic sites and were removed from subsequent analyses. Sequence divergence, as measured with Kimura two-parameter algorithm, ranged from 0.000% to 0.262%, 0.000% to 0.257%, and 0.000% to 0.559% for *atp*B-*rbc*L, *trn*L-*trn*F, and *psb*A-*trn*H, respectively. Sequences of the three spacers conformed to the expectation of neutrality by Tajima's criterion (*atp*B-*rbc*L:*D *= -1.53470, 0.10 > *P *> 0.05; *trn*L-*trn*F:*D *= -1.31009, *P *> 0.10; and *psb*A-*trn*H:*D *= -1.38479, *P *> 0.10) and Fu and Li's criterion (*atp*B-*rbc*L: D* = -1.66525, *P *> 0.10; F* = -1.79736, *P *> 0.10; *trn*L-*trn*F: D* = -1.40980, *P *> 0.10; F* = -1.51361, *P *> 0.10; and *psb*A-*trn*H: D* = -1.28584, *P *> 0.10; F* = -1.48316, *P *> 0.10). The pooled sequences of the three spacers also complied with the expectation of neutrality (*D *= -0.90417, *P *> 0.10; D* = -1.33584, *P *> 0.10; F* = -1.40744, *P *> 0.10). The sequences of nine *atp*B-*rbc*L, eight *trn*L-*trn*F, and thirteen *psb*A-*trn*H haplotypes have been deposited in GenBank databases [GenBank: GQ374124-GQ374155]. Thirty-two haplotypes of *S. baicalensis *(HapA-Y and Hap1-7) and 2 haplotypes of *S. rehderiana *(HapZ1-Z2) were identified when *atp*B-*rbc*L, *trn*L-*trn*F, and *psb*A-*trn*H sequences were combined (Table 2).

**Table 2:** Variable sites of the aligned sequences of three chloroplast DNA fragments in 32 haplotypes of *Scutellaria baicalensis *and 2 haplotypes of *Scutellaria rehderiana*.

Haplotype	Nucleotide position																																	
	
	*atp*B-*rbc*L													*trn*L-*trn*F							*psb*A-*trn*H													
	
	60	193	195	202	203	214	310	381	503	507	603	656	723	22	177	264	265	463	484	726	48	73	151	188	212	236	260	295	326	366	379	435	447	451
HapA	C	C	T	T	-	-	C	G	A	C	T	G	T	-	-	A	-	-	C	C	C	G	-	-	-	-	-	-	-	G	§	-	T	A
HapB	C	C	T	T	-	-	G	G	A	C	T	G	T	-	-	A	-	-	C	A	C	G	-	-	-	-	-	-	A	G	§	-	T	T
HapC	C	C	T	T	-	-	C	G	A	C	T	G	T	-	-	A	-	-	C	A	C	G	-	-	-	-	-	-	A	G	§	-	T	A
HapD	C	C	T	T	-	-	C	G	A	C	T	G	G	-	-	A	-	-	C	A	C	G	-	-	-	-	-	-	A	G	§	-	T	T
HapE	C	C	T	T	-	-	C	G	A	C	T	G	T	-	-	-	-	-	C	A	C	G	-	-	-	-	-	-	A	G	§	-	T	A
HapF	C	C	T	T	-	-	C	G	A	C	T	G	T	-	-	A	-	-	C	A	C	G	-	¶	¶	-	-	-	A	G	§	-	T	T
HapG	C	C	T	T	-	-	C	G	A	C	T	G	T	-	-	A	-	-	C	A	C	G	-	-	-	-	-	-	A	G	§	-	T	T
HapH	C	C	T	T	-	-	C	G	A	C	T	G	T	-	-	A	-	-	C	A	C	G	-	¶	¶	¶	¶	-	A	G	§	-	T	T
HapI	C	C	T	T	-	-	C	G	A	C	T	G	T	-	-	A	-	-	C	A	C	G	-	-	-	-	-	-	A	A	§	-	T	T
hapJ	C	C	T	T	-	-	C	G	A	C	T	G	T	-	-	A	-	-	C	A	C	G	-	¶	-	-	-	-	A	G	§	-	T	T
HapK	C	C	T	T	-	-	C	G	C	C	T	G	T	-	-	A	-	-	C	A	C	G	-	-	-	-	-	-	A	G	§	-	T	A
HapL	C	C	T	T	-	-	G	G	A	C	T	G	T	-	-	A	-	-	C	A	C	G	-	-	-	-	-	-	A	A	§	-	T	T
HapM	C	C	T	T	-	-	G	G	A	C	T	G	T	-	-	-	-	-	A	A	C	G	-	-	-	-	-	-	A	G	§	-	G	T
HapN	C	C	T	T	-	-	C	G	A	C	T	G	T	-	-	A	-	A	C	A	C	G	-	-	-	-	-	-	A	G	§	-	T	T
HapO	C	C	T	T	-	-	G	G	A	C	T	G	T	-	-	A	-	-	C	A	C	G	-	-	-	-	-	-	A	G	§	-	T	T
HapP	C	C	T	T	-	-	C	G	A	C	T	G	T	-	T	A	-	-	C	A	C	G	-	-	-	-	-	-	A	G	§	-	T	T
HapQ	C	C	T	T	-	-	C	G	A	C	T	G	T	-	-	A	-	-	C	A	C	G	-	-	-	-	-	-	A	G	§	-	G	T
HapR	C	C	T	T	-	T	C	G	A	C	T	G	T	-	-	A	-	-	C	A	C	G	-	-	-	-	-	-	A	G	§	-	T	A
HapS	C	C	T	T	-	-	G	G	A	C	T	G	T	-	-	A	-	-	C	A	C	G	-	-	-	-	-	-	A	G	§	-	T	A
HapT	C	C	T	T	-	-	C	G	A	C	T	G	T	-	-	-	-	-	A	A	C	G	-	-	-	-	-	-	A	G	§	-	T	T
HapU	C	C	T	T	-	-	C	G	A	C	T	G	T	-	-	A	-	-	C	A	C	G	#	-	-	-	-	-	A	G	§	-	T	T
HapV	C	C	T	-	-	-	C	G	A	C	T	G	T	-	-	A	-	-	C	A	C	G	-	-	-	-	-	-	A	G	§	-	T	T
HapW	C	C	T	T	-	-	C	G	A	C	T	G	T	-	-	-	-	-	A	A	C	G	-	-	-	-	-	-	A	G	§	-	G	T
HapX	C	C	T	T	-	-	G	G	A	C	T	G	T	-	-	-	-	-	A	A	C	G	-	-	-	-	-	-	A	G	§	-	T	T
HapY	C	C	T	T	-	-	C	G	A	C	T	G	T	-	-	-	-	-	A	A	C	G	-	-	-	-	-	-	A	A	§	-	T	T
HapZ1	T	C	C	T	-	T	C	A	A	T	C	A	T	-	-	-	-	-	A	A	C	T	-	-	-	-	-	-	A	G	-	£	G	T
HapZ2	T	C	C	T	-	T	C	A	A	T	C	A	T	-	-	A	-	-	C	A	C	T	-	-	-	-	-	-	A	G	-	£	G	T
Hap1	C	C	T	T	-	-	C	G	A	C	T	G	T	-	-	A	A	-	C	A	C	G	-	-	-	-	-	-	A	G	§	-	T	T
Hap2	C	T	T	T	-	-	C	G	A	C	T	G	T	-	-	A	-	-	C	A	C	G	-	-	-	-	-	-	A	G	§	-	T	T
Hap3	C	C	T	T	-	-	G	G	A	C	T	G	T	-	-	A	-	-	C	A	T	G	-	-	-	-	-	-	A	G	§	-	T	T
Hap4	C	C	T	T	-	-	G	G	A	C	T	G	T	*	-	A	-	-	C	A	C	G	-	-	-	-	-	-	A	G	§	-	T	T
Hap5	C	C	T	T	-	-	C	G	A	C	T	G	T	*	-	A	-	-	C	A	C	G	-	-	-	-	-	-	A	A	§	-	T	T
Hap6	C	C	T	T	T	-	C	G	A	C	T	G	T	-	-	A	-	-	C	A	C	G	-	-	-	-	-	-	A	A	§	-	T	T
Hap7	C	C	T	T	-	-	G	G	A	C	T	G	T	-	-	A	-	-	C	A	C	G	-	-	-	-	-	‡	A	G	§	-	T	T

HapA-Y and Hap1-7 are haplotypes of *Scutellaria baicalensis*; HapZ1-Z2 are haplotypes of *Scutellaria rehderiana*.

*: CTGAAAAC; #: TTAGTAGTCTTTC; ¶: CTAGACTTATTTCTTTCCATTAAG;

‡: TTCCATTAAGAATAAATAAAG; §: AAAATT; £: GAAAAAAATA.

### Haplotype network and distribution

**Figure 2:**
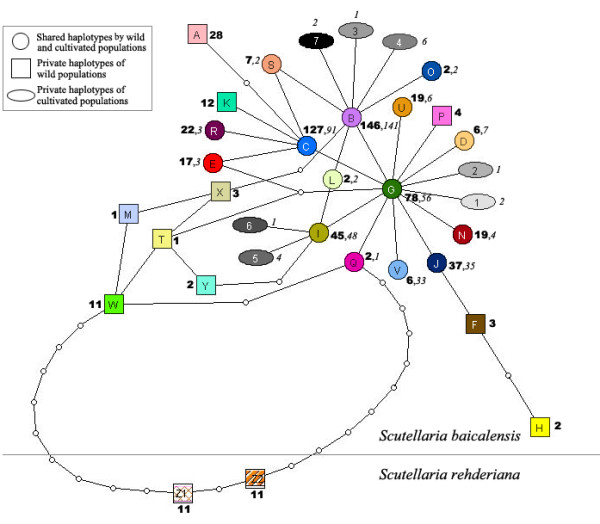
**Nested cladogram of 32 chloroplast haplotypes in *Scutellaria baicalensis *and two haplotypes in *Scutellaria rehderiana***. The color of haplotypes refers to Fig. 1. The bold and italic numbers besides haplotypes represent the number of wild and cultivated individuals with certain haplotype, respectively. Open small circles represent inferred interior nodes that were absent in the samples. Each branch indicates one mutation.

In the haplotype network (Fig. 2), the 32 haplotypes of *S. baicalensis *and 2 haplotypes of *S. rehderiana *differed by at least 10 mutations. The genealogical structure of 32 haplotypes of *S. baicalensis *presented a shallow gene tree with three obvious centers: HapG, HapB and HapC, which connected to other 10, 6, and 4 haplotypes by just one mutation, respectively. Additionally, both HapB and HapC connected to HapG by one mutation. Exceptionally, several haplotypes detected only in wild populations were relatively remote to the three centers of the shallow gene tree, such as HapA, H, M, T, W, X, and Y. The haplotypes of cultivated *S. baicalensis *(symbolized by ovals and circles) evenly distributed across the haplotype network, not showing any clusters.

Haplotype frequencies in each population and geographical distribution are presented in Fig. 1, Additional file 1, and 2. By visual inspection of Fig. 1, the haplotype distributions of wild and cultivated populations were in sharp contrast. The most noticeable pattern was that haplotypes among wild populations were much more structured than those among cultivated populations. For example, HapG in wild populations (WTW, LCW, LPW, CD1W, SXW, and HXW) was mainly restricted to the central range of this species, whereas this haplotype occurred in 17 of 22 cultivated populations across the whole range. Another conspicuous phenomenon was that many wild populations (10 out of 28) were fixed by one unique haplotype, but in cultivated populations, only SXC was fixed by one haplotype (i.e., most cultivated populations characterized by multiple haplotypes).

### Genetic diversity and genetic structure

**Figure 3:**
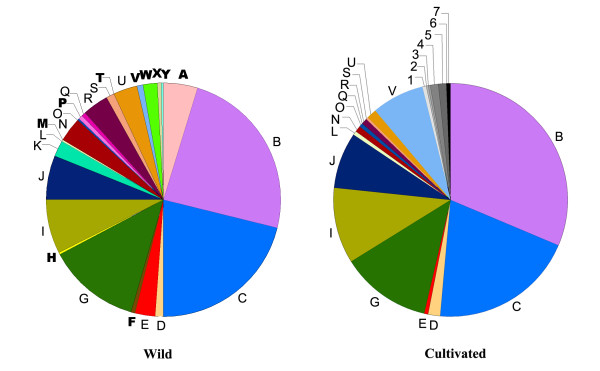
**The relative proportions of different chloroplast haplotypes found in wild and cultivated *Scutellaria baicalensis***. The color of haplotypes refers to Fig. 1. Twenty-five haplotypes were found in wild populations and twenty-two haplotypes were recovered in cultivated populations. Fifteen haplotypes (thin letters) are shared by wild and cultivated groups. Ten (bold letters) and seven (numbers 1-7) haplotypes are specific to wild and cultivated groups, respectively.

Of the 32 haplotypes detected in *S. baicalensis*, 25 (78% of the total number of haplotypes) were recovered in wild populations and 22 (69% of the total number of haplotypes) were carried by cultivated individuals (Fig. 3, Table 3). Fifteen haplotypes (47% of the total number of haplotypes) were shared by wild and cultivated populations (Fig. 3). Ten haplotypes were found in wild populations but not in cultivated ones, and seven were found exclusively in cultivated populations (Fig. 3). By comparison, the number of haplotypes and the relative abundance of each haplotype have slightly changed under the anthropogenic influence during the course of cultivation (Fig. 3, see Additional file 1, 2). However, the change of haplotype frequencies between wild and cultivated groups was not significant by *x*^2 ^test (*P *= 0.733; Table 3), because cultivated and wild groups only differed in rare haplotypes (Fig. 3). As indicated by the haplotype frequencies within population (Fig. 1, see Additional file 1, 2), the within-population diversity (*h*_S _= 0.649) of cultivated *S. baicalensis *was significantly higher than that (*h*_S _= 0.265) of wild Huang-qin (*P *< 0.001, Wilcoxon two-group test; Table 3), although the total haplotype diversity was similar between cultivated and wild groups (*h*_T _= 0.832 in cultivated vs *h*_T _= 0.888 in wild, *P *> 0.05, Wilcoxon two-group test; Table 3).

**Table 3:** Comparisons of genetic diversity and genetic structure between wild and cultivated *Scutellaria baicalensis *populations.

Parameter	wild	cultivated	*P*
Number of haplotype	25	22	0.733†
Total diversity, *h*_T_	0.888 (0.0287)	0.832 (0.0234)	> 0.05‡
Within-population diversity, *h*_S_	0.265 (0.0526)	0.649 (0.0425)	**< 0.001‡**
Population differentiation, *G*_ST_	0.701 (0.0594)	0.220 (0.0449)	**< 0.001‡**

Parameters of population subdivision are followed by standard error in parentheses.

Statistically significant comparisons are highlighted in bold type.

†: *x*^2 ^test; ‡: Wilcoxon two-group test.

**Figure 4:**
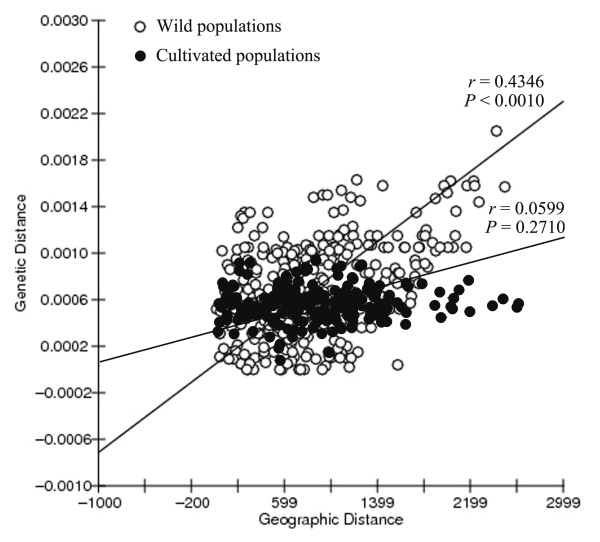
**Scatterplots of genetic distances (Kimura 2-parameter distance) against geographical distances (kilometres) separating each pairwise combination of populations within wild and cultivated *Scutellaria baicalensis***.

Consistent with the haplotype distribution (Fig. 1), population subdivision of wild populations (*G*_ST _= 0.701) was significantly higher than that of the cultivated (*G*_ST _= 0.220; *P *< 0.001, Wilcoxon two-group test; Table 3). Mantel test analyses showed that genetic distance was significantly correlated with geographical distance (*r = *0.4346, *P *< 0.0010; Fig. 4) in wild populations, however, this pattern was not recovered in cultivated populations (*r = *0.0599, *P *= 0.2710; Fig. 4). AMOVA analysis showed that little genetic variation occurred between cultivated and wild populations (0.09%, *P *< 0.001), and most genetic variance were among populations (56.61%) and within populations (43.30%; Table 4). The genetic differentiation (*F*_ST _= 0.022) between the cultivated and wild groups calculated by DNASP 4.00 was well consistent with the result of AMOVA analysis.

**Table 4:** Hierarchical analysis of molecular variance for 50 populations of *Scutellaria baicalensis*.

Hierarchical level	Deg. of freedom	Sum of squares	Variance components	Percentage of variance	*F*-statistics	*P*-value*
Among groups	1	7.740	0.00055	0.09	*F*_CT _= 0.00091	< 0.001
Among populations within groups	48	356.792	0.34067	56.61	*F*_ST _= 0.56698	< 0.001
Within populations	1003	261.380	0.26060	43.30	*F*sc = 0.56659	< 0.001

All populations are partitioned into cultivated and wild groups.

*: Significance calculated using 1023 permutations.

## Discussion

### Slight reduction of genetic diversity and little change of genetic composition in cultivated *S. baicalensis*

Cultivation of wild plants always produces genetic bottlenecks and thus results in loss of genetic diversity due to founder effects and unconscious or conscious selections [8]. For example, only 23% of the total chloroplast haplotypes detected in *Oryza rufipogon *and *O. sativa *were recovered in the cultivated rice [29]. However, concerns about the impacts of cultivation bottlenecks are mostly concentrated on economically valuable crops with long domestication history, little is known about the genetic impacts of recent cultivation on medicinal plants. In this study, slight reduction of genetic diversity and little change of genetic composition during the cultivation of *S. baicalensis *were revealed (Fig. 3, Table 3). These results are in sharp contrast to most crop species with long cultivation histories and may be explained by the large initial population size of cultivated *S. baicalensis*, the short cultivation history, and weak artificial selection pressure.

The strength of genetic bottleneck during cultivation is determined by two interacting factors: the size of the bottlenecked population and the bottleneck's duration [8,45]. Intuitively, cultivated plants of multiple origins (multiple places and multiple events) would hold larger population size and thus most likely maintain greater genetic diversity [46]. This point has been supported by a handful of empirical studies (e.g., *Magnolia officinalis *var. *biloba *[16]; einkorn [47]; *Spondias purpurea *[6]), although artificial selection and founder-event-induced genetic drift over the course of subsequent domestication are capable of reducing genetic diversity in multiple-origin plants (e.g. [29,44]). In this study, different lines of evidence support that cultivated *S. baicalensis *could have originated from different places for multiple times, resulting in large initial population size during cultivation. First, a large proportion of haplotypes (47% of the total number of haplotypes) were found in both wild and cultivated populations and these shared haplotypes were recovered from different wild populations (Fig. 1, 3). This even distribution pattern of shared alleles in cultivated and wild populations strongly suggests that genetically distinct individuals from different geographic regions were taken into cultivation and subsequently distributed by humans [44,48]. Second, no cluster of cultivated haplotypes was observed in the haplotype network (Fig. 2), indicating that cultivated *S. baicalensis *is of multiple origins because crops of single origin often form a monophyletic clade in a phylogenetic tree that includes wild progenitors [46].

According to the tremendous literature of traditional Chinese medicine, Huang-qin was rarely cultivated until the foundation of the People's Republic of China in 1949 [27]. However, over the past several decades, the wild resource of this species declined rapidly because of over-exploitation. To meet the increasing demand for the root of this species, large-scale cultivation began since 1958 [27]. Such a short cultivation history, in combination with the large initial population size, may be unlikely to produce a strong genetic bottleneck, resulting in significant reduction of genetic diversity and change in genetic composition of cultivated *S. baicalensis*. Other population genetic studies on recently-cultivated Chinese medicinal plants also suggested that high genetic diversity in cultivated populations is due to their slight cultivation bottlenecks [15-17], which provides genetic raw materials for breeding new varieties of medicinal plants.

Apart from demographic factors, selection is another important element in shaping the genetic diversity pattern of cultivated species. Artificial selection for desired traits can decrease genetic diversity of target loci and their linked loci beyond that caused by the bottlenecks [8]. The fact that only few cultivars of *S. baicalensis *have been officially registered in China [49] suggests that artificial selection on this cultivated medicinal herb is weak, which resulting in only slight reduction of genetic diversity during cultivation. In addition, the relaxation in selective constraint under cultivated conditions might also contribute to the high genetic diversity within cultivated *S. baicalensis *populations because relaxation of selective constraint allow the accumulation of mutations that may otherwise be deleterious in nature [50,51]. However, considering the short cultivation history of *S. baicalensis*, the relaxation of selective constraint might play limited role in molding the genetic diversity pattern of cultivated Huang-qin.

### Extensive seed exchange among cultivated *S. baicalensis *populations

Cultivation impacts not only the amount of genetic variation contained in cultivated populations but also the structure of this variation [48,52,53]. In this study, we found that the cultivated *S. baicalensis *populations displayed a lower proportion of genetic variation among populations (*G*_ST _= 0.220) than wild populations (*G*_ST _= 0.701). In addition, Mantel test analyses showed that the effect of geographical isolation on genetic structure was significant in wild populations (*r = *0.4346, *P *< 0.0010; Fig. 4), but not in cultivated populations (*r = *0.0599, *P *= 0.2710; Fig. 4). These results suggest that cultivation has imposed a profound impact on the genetic structure of cultivated *S. baicalensis*. However, these findings run contrary to the results of Hamrick and Godt [53], which reported that the mean value of genetic differentiation among crop species (*G*_ST _= 0.339) is higher than that of noncrop species (*G*_ST _= 0.212).

Two possible reasons may account for the unusual pattern revealed in *S. baicalensis*. First, there has been insufficient time for artificial selection and breeding to exert influence on the genetic divergence of cultivated *S. baicalensis*. The crop species (i.e. *Zea mays*, *Hordeum vulgare*, etc.) summarized in the review of Hamrick and Godt [53] almost have long histories of cultivation. Long-term artificial selection and inbreeding within cultivars imposed by plant breeding could have resulted in the genetic variation occurring generally among cultivars [52,54,55]. However, for *S. baicalensis*, due to its short cultivation history, artificial selection and breeding cultivars are unlikely to promote the genetic differentiation of cultivated populations as suggested by few *S. baicalensis *cultivars in China [49]. This fact indicates that standard industrial cultivation, a practice possibly imposing high artificial selection pressure on medicinal herbs, has not been established for *S. baicalensis *[4].

Second, homogenization among cultivated populations as well as high within-population diversity could have been facilitated by extensive seed exchange among different geographic locations due to highly developed transportation and commercial markets of modern times. A study of pepino (*Solanum muricatum*), a herbaceous Andean domesticate, suggested that human interchange among communities and countries in the past 50 years or so has considerably weakened the geographic differentiation of genetic diversity of cultivated pepinos [9]. For Chinese cultivated medicinal plants, such as *Coptis chinensis*, *Magnolia officinalis*, the bulking and mixing of seeds from different geographic locations are the common practice, which produced lower genetic differentiation among cultivated populations [16,17]. However, the genetic markers (ISSR and AFLP) used in those studies are biparentally transmitted, incapable of ruling out the effects of seed flow on genetic structure. In this study, by using maternally inherited chloroplast markers which can trace seed flow among populations [6,29], we found that the effects of geographical isolation were significant in wild populations (*r = *0.4346, *P *< 0.0010; Fig. 4) but disappeared in cultivated populations (*r = *0.0599, *P *= 0.2710; Fig. 4). In addition, the haplotype distribution maps also visualize that common haplotypes are much more widespread in cultivated *S. baicalensis *than in wild populations. For example, HapG in wild populations (WTW, LCW, LPW, CD1W, SXW, and HXW) is mainly restricted to the central range of this species, whereas this haplotype occurred in 17 of 22 cultivated populations (Fig. 1). These patterns explicitly demonstrate that homogenization among cultivated populations and high within-population diversity should be mostly due to seed exchange mediated by human activities under cultivation.

### Conservation implications of *S. baicalensis *cultivation

It has long been argued that traditional agriculture can serve as an important reservoir of genetic variability [5,56]. For example, by taking the jocote (*Spondias purpurea*), a small tree that bears fruit similar to tiny mongo in Mesoamerica, out of its natural, wild habitats and planting them in yards and other means of cultivation, farmers in the Mesoamerican region have helped to preserve the jocote's diversity [48]. Likewise, traditional cultivations is also an effective way for the maintenance and conservation of gene pools of medicinal plants, as evidenced by the studies of Guo *et al*. [15] and He *et al*. [16]. According to our investigation, the cultivation practices for *S. baicalensis *are also in traditional ways. Usually, farmers collected the seeds of *S. baicalensis *directly from local wild resources and then cultivated in the field. Sometimes, the germplasm can be dispersed to other places by their relatives or friends or by sale in the markets. Slight reduction of genetic diversity and little change of genetic composition in cultivated *S. baicalensis*, as well as a few specific haplotypes of cultivated group, confirm the effectiveness of saving plant diversity through local cultivation. This study further suggests that bringing wild medicinal plants into cultivation from different locations may help to widen the genetic background of cultivated populations, which will be beneficial for the sustainable utilization of natural resources.

Although cultivation is an effective strategy for preserving genetic resources of *S. baicalensis*, the wild resources still need to be protected *in situ*. This study did not find significant reduction of genetic diversity and apparent change of genetic composition in cultivated *S. baicalensis*, however, this pattern reflects mainly the changes of common haplotypes rather than rare alleles. In fact, 10 out of 25 haplotypes within wild populations have lost during the course of *S. baicalensis *cultivation. The results conform to the theoretical expectation that bottlenecks of short duration may have little effect on heterozygosity (here represented by haplotype diversity, *h*_T_) but will reduce severely the number of rare alleles [57]. Rare alleles are often considered a minor element in genetic conservation programs and yet they can be very important for long-term evolution or to meet new breeding objectives such as resistance to introduced insects or diseases [57]. It is therefore desirable to maintain rare alleles of *S. baicalensis *through *in situ *conservation of wild populations.

Seed exchange during the course of *S. baicalensis *cultivation exerts a significant impact on the genetic architecture of cultivated *S. baicalensis *and may also have profound implications for the conservation and utility of *S. baicalensis *germplasm. Genetic mixture induced by extensive seed exchange means that a fraction of cultivated populations may be sufficient to preserve most genetic variation of *S. baicalensis*. This will undoubtedly facilitate the conservation of genetic diversity of *S. baicalensis *through cultivation. However, numerous theoretical and empirical studies have suggested that genetic mixture of populations that are adapted to different local conditions could result in outbreeding depression, the reduction in fitness caused by the breakdown of coadapted gene complexes [58]. Extensive seed exchange may increase the risk of maladaptation and reduced growth or fertility resulting from maladaptation could reduce the success of cultivation projects and jeopardize the long-term survival of wild populations [59]. The evolutionary and practical consequences of extensive seed exchange of *S. baicalensis *are unknown so far, more studies are crucially needed to monitor the potentially negative impacts of seed exchange on the adaptation of wild populations and the growth of cultivated populations.

## Conclusions

Facing the rapidly growing demands for medicinal plants, domestic cultivation is a viable and long-term way of protecting wild medicinal plant resources [3]. However, this study indicates that bringing a species into cultivation may impose profound impacts on genetic diversity patterns and even the evolutionary potentials of medicinal plants. Although the total genetic diversity maintained in cultivated *S. baicalensis *is comparable to wild populations due to the large initial population size and the short cultivation history, substantial rare alleles have lost and extensive seed exchange has caused the homogenization of cultivated populations during the course of cultivation. This study not only provides baseline data for preserving genetic resource of *S. baicalensis *through conservation-by-cultivation approach, but also represents a paradigm for evaluating the genetic impacts of recent cultivation on medicinal plants, which may be instructive to future cultivation projects of traditional Chinese medicinal plants.

## Authors' contributions

L-QH conceived of the study and revised the manuscript. Q-JY performed sample collection, molecular experiment, data analysis and drafted the manuscript. Z-YZ contributed to the conception of the study, the writing and revision of the manuscript. JH participated in DNA extraction, PCR and sequencing. L-PG helped to coordinate the study and finish statistical tests. A-JS carried out partial sample collection. All authors read and approved the final manuscript.

## Authors' informations

Prof. Lu-Qi Huang specializes in molecular pharmaceutics and the taxonomy of some systematically uncertain taxa in Chinese herbal medicine, such as *Trichosanthes*. Dr. Qing-Jun Yuan is interested in conservation genetics and domestication of cultivated medicinal plants. Prof. Zhi-Yong Zhang is working on molecular phylogeography and molecular systematics in the subtropical plants. Juan Hu is a master student studying the application of molecular markers in authentication of Chinese Materia Medica. Prof. Lan-Ping Guo and Ai-Juan Shao are with special concerns about the sustainable utilization and conservation of natural medicinal resources.

## Supplementary Material

Additional file 1 Chloroplast haplotype frequencies in 28 wild populations of *Scutellaria baicalensis *and 1 wild population of *Scutellaria rehderiana*. Click here for file

Additional file 2 Chloroplast haplotype frequencies in 22 cultivated populations of *Scutellaria baicalensis*. Click here for file
