# Supplementary material for: Impacts of recent cultivation on genetic diversity pattern of a medicinal plant, Scutellaria baicalensis (Lamiaceae)
Source: BMC Genet. 2010 Apr 29;11:29. doi: 10.1186/1471-2156-11-29 (PMC2877650; doi:10.1186/1471-2156-11-29)
Supplement: Additional file 1 — Chloroplast haplotype frequencies in 28 wild populations of Scutellaria baicalensis and 1 wild population of Scutellaria rehderiana. [file 1471-2156-11-29-S1.DOC]

**Additional file [1](http://www.pubmedcentral.nih.gov/articlerender.fcgi?artid=2628941#S1)** Chloroplast haplotype frequencies in 28 wild populations of *Scutellaria baicalensis* and 1 wild population of *Scutellaria rehderiana*.

| P | N | cpDNA haplotype | | | | | | | | | | | | | | | | | | | | | | | | | | |
| --- | --- | --- | --- | --- | --- | --- | --- | --- | --- | --- | --- | --- | --- | --- | --- | --- | --- | --- | --- | --- | --- | --- | --- | --- | --- | --- | --- | --- |
|  |  | A | B | C | D | E | F | G | H | I | J | K | L | M | N | O | P | Q | R | S | T | U | V | W | X | Y | Z1 | Z2 |
| *Scutellaria baicalensis* | | | | | | | | | | | | | | | | | | | | | | | | | | | | |
| EGW | 20 | 4 |  | 16 |  |  |  |  |  |  |  |  |  |  |  |  |  |  |  |  |  |  |  |  |  |  |  |  |
| LXW | 24 |  | 5 |  |  |  |  |  |  |  |  |  |  |  |  |  |  |  |  |  |  | 19 |  |  |  |  |  |  |
| KKW | 22 |  | 18 |  |  |  |  |  |  |  |  |  |  |  |  |  |  |  |  | 4 |  |  |  |  |  |  |  |  |
| GYW | 24 |  |  |  |  |  |  |  |  |  | 2 |  |  |  |  |  |  |  | 22 |  |  |  |  |  |  |  |  |  |
| HMW | 24 | 24 |  |  |  |  |  |  |  |  |  |  |  |  |  |  |  |  |  |  |  |  |  |  |  |  |  |  |
| DMW | 24 |  | 7 | 16 | 1 |  |  |  |  |  |  |  |  |  |  |  |  |  |  |  |  |  |  |  |  |  |  |  |
| BCW | 19 |  | 1 | 11 |  | 1 | 3 | 1 | 2 |  |  |  |  |  |  |  |  |  |  |  |  |  |  |  |  |  |  |  |
| YJW | 21 |  |  | 21 |  |  |  |  |  |  |  |  |  |  |  |  |  |  |  |  |  |  |  |  |  |  |  |  |
| JCW | 22 |  | 3 | 6 | 1 |  |  | 3 |  |  | 1 |  |  |  |  | 2 | 4 | 1 |  |  |  |  | 1 |  |  |  |  |  |
| JZW | 20 |  |  |  |  |  |  |  |  |  | 8 | 12 |  |  |  |  |  |  |  |  |  |  |  |  |  |  |  |  |
| CD1W | 18 |  |  | 13 |  |  |  | 5 |  |  |  |  |  |  |  |  |  |  |  |  |  |  |  |  |  |  |  |  |
| CD2W | 23 |  | 23 |  |  |  |  |  |  |  |  |  |  |  |  |  |  |  |  |  |  |  |  |  |  |  |  |  |
| KCW | 17 |  |  |  |  | 16 |  |  |  |  |  |  |  |  |  |  |  |  |  | 1 |  |  |  |  |  |  |  |  |
| LPW | 18 |  |  | 5 |  |  |  | 12 |  |  |  |  |  |  |  |  |  |  |  |  |  |  | 1 |  |  |  |  |  |
| CCW | 22 |  |  | 21 |  |  |  | 1 |  |  |  |  |  |  |  |  |  |  |  |  |  |  |  |  |  |  |  |  |
| YQ1W | 19 |  |  |  |  |  |  |  |  |  |  |  |  |  | 19 |  |  |  |  |  |  |  |  |  |  |  |  |  |
| YQ2W | 22 |  |  | 18 |  |  |  |  |  |  | 2 |  |  |  |  |  |  |  |  | 2 |  |  |  |  |  |  |  |  |
| YTW | 24 |  |  |  |  |  |  |  |  |  | 24 |  |  |  |  |  |  |  |  |  |  |  |  |  |  |  |  |  |
| HXW | 22 |  | 3 |  | 4 |  |  | 5 |  | 6 |  |  |  |  |  |  |  |  |  |  |  |  | 4 |  |  |  |  |  |
| SXW | 19 |  |  |  |  |  |  | 6 |  | 12 |  |  | 1 |  |  |  |  |  |  |  |  |  |  |  |  |  |  |  |
| WTW | 24 |  |  |  |  |  |  | 24 |  |  |  |  |  |  |  |  |  |  |  |  |  |  |  |  |  |  |  |  |
| FYW | 23 |  | 22 |  |  |  |  |  |  | 1 |  |  |  |  |  |  |  |  |  |  |  |  |  |  |  |  |  |  |
| LCW | 21 |  |  |  |  |  |  | 21 |  |  |  |  |  |  |  |  |  |  |  |  |  |  |  |  |  |  |  |  |
| JXW | 23 |  | 23 |  |  |  |  |  |  |  |  |  |  |  |  |  |  |  |  |  |  |  |  |  |  |  |  |  |
| HLW | 24 |  | 21 |  |  |  |  |  |  | 1 |  |  |  |  |  |  |  |  |  |  |  |  |  |  | 2 |  |  |  |
| SYW | 19 |  |  |  |  |  |  |  |  | 1 |  |  | 1 | 1 |  |  |  | 1 |  |  | 1 |  |  | 11 | 1 | 2 |  |  |
| TBW | 24 |  |  |  |  |  |  |  |  | 24 |  |  |  |  |  |  |  |  |  |  |  |  |  |  |  |  |  |  |
| HSW | 20 |  | 20 |  |  |  |  |  |  |  |  |  |  |  |  |  |  |  |  |  |  |  |  |  |  |  |  |  |
| Total | 602 | 28 | 146 | 127 | 6 | 17 | 3 | 78 | 2 | 45 | 37 | 12 | 2 | 1 | 19 | 2 | 4 | 2 | 22 | 7 | 1 | 19 | 6 | 11 | 3 | 2 |  |  |
| *Scutellaria rehderiana* | | | | | | | | | | | | | | | | | | | | | | | | | | | | |
| WYW | 22 |  |  |  |  |  |  |  |  |  |  |  |  |  |  |  |  |  |  |  |  |  |  |  |  |  | 11 | 11 |

Abbreviations: P, population code; N, number of sampled individuals.
