# Supplementary material for: Impacts of recent cultivation on genetic diversity pattern of a medicinal plant, Scutellaria baicalensis (Lamiaceae)
Source: BMC Genet. 2010 Apr 29;11:29. doi: 10.1186/1471-2156-11-29 (PMC2877650; doi:10.1186/1471-2156-11-29)
Supplement: Additional file 2 — Chloroplast haplotype frequencies in 22 cultivated populations of Scutellaria baicalensis. [file 1471-2156-11-29-S2.DOC]

**Additional file [2](http://www.pubmedcentral.nih.gov/articlerender.fcgi?artid=2628941#S1)** Chloroplast haplotype frequencies in 22 cultivated populations of *Scutellaria baicalensis*.

| P | N | cpDNA haplotype | | | | | | | | | | | | | | | | | | | | | |
| --- | --- | --- | --- | --- | --- | --- | --- | --- | --- | --- | --- | --- | --- | --- | --- | --- | --- | --- | --- | --- | --- | --- | --- |
|  |  | B | C | D | E | G | I | J | L | N | O | Q | R | S | U | V | 1 | 2 | 3 | 4 | 5 | 6 | 7 |
| KQLC | 20 | 2 | 15 |  |  | 2 |  |  |  |  |  |  |  |  | 1 |  |  |  |  |  |  |  |  |
| KQIC | 24 | 4 | 17 |  |  |  |  |  |  |  |  |  |  |  | 3 |  |  |  |  |  |  |  |  |
| LBC | 17 | 2 | 4 |  |  | 3 | 5 |  |  |  |  |  |  |  | 2 |  |  |  |  |  | 1 |  |  |
| CCC | 19 | 6 | 4 | 1 | 1 | 3 | 2 | 2 |  |  |  |  |  |  |  |  |  |  |  |  |  |  |  |
| YXC | 22 | 3 | 4 |  | 1 | 1 |  | 13 |  |  |  |  |  |  |  |  |  |  |  |  |  |  |  |
| CD1C | 17 | 5 | 2 |  |  | 4 | 1 | 2 |  |  | 1 | 1 |  |  |  |  |  |  |  | 1 |  |  |  |
| CD2C | 21 | 15 |  |  |  | 3 |  | 2 |  |  |  |  |  |  |  | 1 |  |  |  |  |  |  |  |
| KCC | 19 | 8 | 1 | 1 | 1 | 1 | 4 |  |  |  |  |  |  |  |  | 1 | 2 |  |  |  |  |  |  |
| LPC | 20 | 11 | 6 | 1 |  | 2 |  |  |  |  |  |  |  |  |  |  |  |  |  |  |  |  |  |
| YQC | 23 |  |  | 2 |  | 7 | 2 |  |  |  |  |  |  |  |  | 12 |  |  |  |  |  |  |  |
| JNC | 20 | 14 |  |  |  |  |  | 3 |  |  | 1 |  |  |  |  |  |  |  |  | 2 |  |  |  |
| JUXC | 19 | 10 | 4 |  |  | 1 |  |  |  |  |  |  |  | 1 |  |  |  |  |  | 3 |  |  |  |
| JRC | 24 | 8 | 3 |  |  | 4 |  | 2 |  |  |  |  |  |  |  | 7 |  |  |  |  |  |  |  |
| SXC | 21 | 21 |  |  |  |  |  |  |  |  |  |  |  |  |  |  |  |  |  |  |  |  |  |
| FXC | 21 |  |  | 2 |  |  | 13 |  | 1 |  |  |  |  |  |  | 4 |  |  |  |  |  | 1 |  |
| FYC | 21 | 8 |  |  |  | 1 | 5 |  | 1 | 1 |  |  |  | 1 |  |  |  | 1 |  |  | 3 |  |  |
| LCC | 22 | 3 |  |  |  | 15 | 3 | 1 |  |  |  |  |  |  |  |  |  |  |  |  |  |  |  |
| JXC | 20 | 5 | 11 |  |  | 1 | 1 |  |  |  |  |  | 1 |  |  |  |  |  | 1 |  |  |  |  |
| HLC | 18 | 4 |  |  |  | 2 | 5 |  |  |  |  |  |  |  |  | 7 |  |  |  |  |  |  |  |
| TBC | 22 | 6 | 10 |  |  | 3 |  | 1 |  | 2 |  |  |  |  |  |  |  |  |  |  |  |  |  |
| WYC | 21 | 4 | 1 |  |  | 3 | 2 | 6 |  |  |  |  | 2 |  |  | 1 |  |  |  |  |  |  | 2 |
| ZXC | 20 | 2 | 9 |  |  |  | 5 | 3 |  | 1 |  |  |  |  |  |  |  |  |  |  |  |  |  |
| Total | 451 | 141 | 91 | 7 | 3 | 56 | 48 | 35 | 2 | 4 | 2 | 1 | 3 | 2 | 6 | 33 | 2 | 1 | 1 | 6 | 4 | 1 | 2 |

Abbreviations: P, population code; N, number of sampled individuals.
